# Detection of Architectural Dysplastic Features from Histopathological Imagery of Oral Mucosa Using Neural Networks

**DOI:** 10.3390/bioengineering12030216

**Published:** 2025-02-20

**Authors:** Watchanan Chantapakul, Sirikanlaya Vetchaporn, Sansanee Auephanwiriyakul, Nipon Theera-Umpon, Ritipong Wongkhuenkaew, Uklid Yeesarapat, Nutchapon Chamusri, Mansuang Wongsapai

**Affiliations:** 1 Biomedical Engineering Institute, and the Biomedical Engineering and Innovation Research Center, Chiang Mai University, Chiang Mai 50200, Thailand; tmwatchanan@gmail.com (W.C.); nipon.t@cmu.ac.th (N.T.-U.); ritipong.w@cmu.ac.th (R.W.); 2 Intercountry Centre for Oral Health, Department of Health, Ministry of Public Health, Chiang Mai 50000, Thailand; sirikanlaya.v@anamai.mail.go.th (S.V.); mansuangdent@yahoo.com (M.W.); 3 Department of Computer Engineering, Faculty of Engineering, Chiang Mai University, Chiang Mai 50200, Thailand; uklid_y@cmu.ac.th; 4 Department of Electrical Engineering, Faculty of Engineering, Chiang Mai University, Chiang Mai 50200, Thailand; 5 Department of Oral Biology and Diagnostic Sciences, Faculty of Dentistry, Chiang Mai University, Chiang Mai 50200, Thailand; nutchapon.c@cmu.ac.th

**Keywords:** bulbous rete ridges, convolutional neural networks, histopathological image, irregular epithelial stratifications, oral epithelial dysplasia

## Abstract

Oral cancer is a serious illness, but it is potentially curable if early detection can be achieved successfully. Oral epithelial dysplasia (OED), which is a precursor to oral squamous cell carcinoma (OSCC), can provide abnormal characteristics to diagnose the risk of developing oral cancer. This paper proposes a neural network architecture for detecting dysplastic features of epithelial architecture, including irregular epithelial stratification and bulbous rete ridges. The different combinations of atrous convolution, batch normalization, global pooling, and dropout are discussed regarding their effects, along with an ablation study. A signature library containing image patches was constructed and utilized to train the models. The best-performing model in the validation set attained an average accuracy of 97.52%. The results of the blind test from the receiver operating characteristic (ROC) curves show that the best model reached the best probability of detection, 0.8571, for irregular epithelial stratifications and 0.8462 for the bulbous rete ridges.

## 1. Introduction

The oral cavity is the first and most important part of the digestive system. Maintaining oral health is necessary but, at the same time, not an easy task. In addition, apart from our behaviors, there are many uncontrollable or unexpected factors that can be detrimental to oral health. People generally worry about cancer more than other serious illnesses. Evidently, according to the World Health Organization (WHO) [1], oral cancer is certainly a serious problem. They provide the incidence estimates of lip and oral cavity cancers worldwide at an estimated 377,713 new cases and 177,757 mortalities which occurred in 2020. On top of that, oral cancer was ranked the 13th most common cancer globally. There are many works [2,3,4,5,6] that have proposed deep learning-based models to classify or detect oral cancer (malignant oral lesion) from photographic images, and this is still an active line of research.

Oral potentially malignant disorders (OPMDs) comprise several oral health conditions affecting the oral mucosa and posing a susceptibility to the increased risk of malignancy development. Some OPMDs encompass oral epithelial dysplasia (OED) [7]. Since oral epithelial dysplasia is the precursor to malignancy, it is critical to identify this condition. To identify OED is to consider architectural and cytological changes at the histological level. Thus, in this case, we are interested in OED, which is a premalignant condition characterized by aberrations in the histological structure of the oral mucosa (the lining inside of the mouth). OED carries the risk of developing into oral squamous cell carcinoma (OSCC), which is one of the most common oral cancers affecting the lips, tongue, gums, and palate. Nevertheless, not all OED progresses to cancer [8]. Simply put, OED is considered a precancerous condition, i.e., a precursor to oral cancer. That is, if we can detect the OED characteristics early, we can appropriately plan the proper treatment.

There are research works involving utilizing artificial intelligent techniques in classifying between oral cancer tissue and normal tissue, such as [9,10,11,12]. However, these works only performed analysis of the cytological atypia and did not include the epithelial architecture. Since the study of artificial intelligence in detecting OPMDs from abnormalities and indicating an abnormal level in the epithelial dysplasia is difficult, there are few studies in dysplasia classification based on handcrafted features [13,14,15,16]. Also, there are limitations. Bashir et al. [13] proposed dysplasia classifiers based on two steps, layer segmentation with the semantic segmentation network and a random forest model with morphometric features. Traditional support vector machine (SVM) and K-nearest neighbor (K-NN) can also be used as classifiers to perform binary classification of OED based on handcrafted image features [9,10]. Although the merit of using these handcrafted features is their explainability, their performances might be inferior to the convolutional neural network-based models that can automatically extract features [13,15]. There are also many endeavors in exploiting deep neural networks such as the works [5,17,18,19,20,21,22].

The aforementioned studies have contributed to the advancement of automated analysis of oral histopathological images, but there remains a need for more accurate and efficient methods for detecting specific dysplastic features. While these approaches have shown promise in OED classification, they often focus on overall dysplasia detection rather than identifying specific architectural features. Our study aims to address this gap by developing a model that can detect and classify individual dysplastic characteristics, such as irregular epithelial stratification and bulbous rete ridges. This granular approach could potentially provide more detailed and actionable information for pathologists and clinicians in assessing OED severity and progression risk.

OEDs can be observed through the lens of various microscopic dysplastic features such as irregular epithelial stratification, bulbous rete ridges (pegs), hyperchromatism, atypical mitotic figures, increased mitosis, pleomorphism, and dyskeratosis. In this study, our focus lies in the first two abnormalities, since they are represented in the form of architectural patterns in lieu of individual malformation. Moreover, dysplasia is abnormal changes in the oral mucosal structure that potentially precede the development of oral cancer.

CNNs have been used in diagnosis/prognosis with several medical images, such as breast cancer detection from mammograms [23,24,25], brain tumor detection from MRI [26,27], etc. [28,29,30,31]. The advantages of using CNN-based models with medical images are similar, that is, that they can be trained without extracting features from images. Since there are filters in the process, local connectivity and the context of the pixels are preserved. Hence, the variability and diversity of the image are kept. However, one of the disadvantages of CNN-based model in medical images is that the training process is computationally expensive. If the testing is carried out on a whole slide image or a large medical image, the computational complexity is still large. For a classification purposes, abnormal classes in any type of medical image dataset are much smaller than that in normal class; hence, there might be an overfitting in the training process.

As mentioned previously, CNNs have been used in the analysis of oral histopathological images [5,17,18,19,20,21,22] but not each dysplasia feature. Dysplasia features can give us more details on OED severity assessment and progression risk better than just stating that each image contains abnormal or normal cells.

Hence, in this paper, we propose a neural network that consists of two parts: (1) a convolutional neural network (CNN) and (2) a fully connected network (FCNN). The main contribution is the components included and explored in designing the best architecture. The regularization methods, batch normalization and dropout are discussed. One of the most important contributions of this work is the best approach for bridging a CNN (the output is feature maps, which have high dimensionality) to an FCNN using global average pooling and its variants, which can drastically reduce the complexity of the model, i.e., the number of required parameters.

Thus, the structure of this paper starts by elaborating upon the oral epithelial dysplasia dataset used, which will provide information about how the images look for both no-dysplasia and architectural dysplasia characteristics. Next, all technical details are laid out in Section 3, including CNNs and their relevant components. Finally, the experimental results that reflect the performances of the models on the signature library and the blind set are explained.

## 2. Oral Epithelial Dysplasia

**Figure 1:**
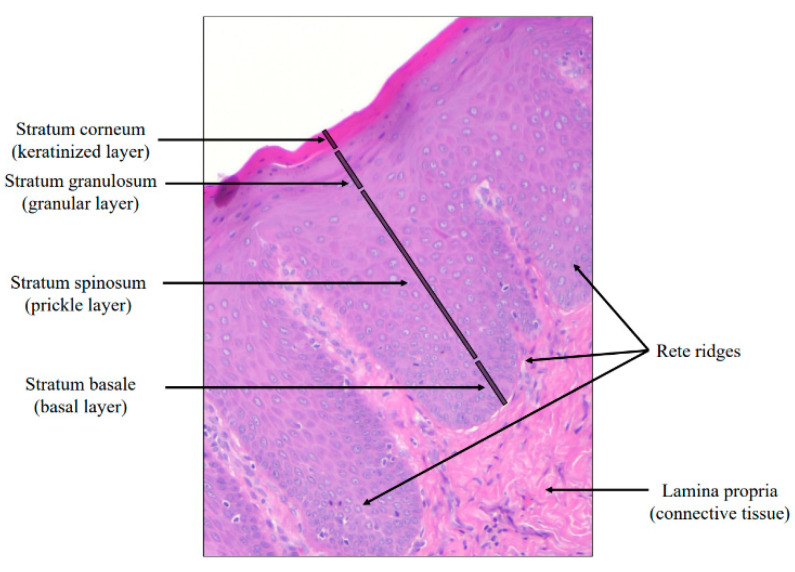
A color image of oral mucosa structure. It is divided into the epithelium and the lamina propria. The epithelium can be broken down further into four layers.

Let us briefly review the knowledge about the oral mucosa (mucosa of the mouth) [32,33]. As depicted in Figure 1, the oral epithelium and the lamina propria constitute oral mucosa. The oral epithelium is composed of four layers including (1) the stratum corneum, (2) the stratum granulosum, (3) the stratum spinosum, and (4) the stratum basale. The stratum basale or basal layer is adjacent to the connective tissue.

### 2.1. Dysplastic Features

Oral epithelial dysplasia (OED) is a precursor condition to squamous cell carcinoma (SCC), which is the most common type of oral cancer. However, OED can develop from oral potentially malignant disorders (OPMDs), but not all of them can progress to SCC. Thus, OED must be differentiated from SCC and those OPMDs which do not contain dysplastic features [7].

OED contains dysplastic features [34], which are cellular abnormalities resulting in loss of cell pattern, cell orientation, cell maturation, and cell function. The abnormalities include both epithelial architecture abnormalities and cytologic atypia. The characteristics of epithelial architecture abnormalities include basal cell crowding and irregular epithelial stratification, reduced intercellular adhesion, bulbous or drop-shaped rete ridges, increased numbers of mitotic figures, abnormal superficial mitoses, and premature keratinization in single cells (dyskeratosis). Meanwhile, the characteristics of cytologic atypia include increased nuclear size, abnormal variations in nuclear shape (nuclear pleomorphism), abnormal variation in cell size (anisocytosis), abnormal variation in cell shape (cellular pleomorphism), increased nuclear–cytoplasmic ratio, atypical mitotic figures, increased number and size of nucleoli, and hyperchromasia.

**Figure 2:**
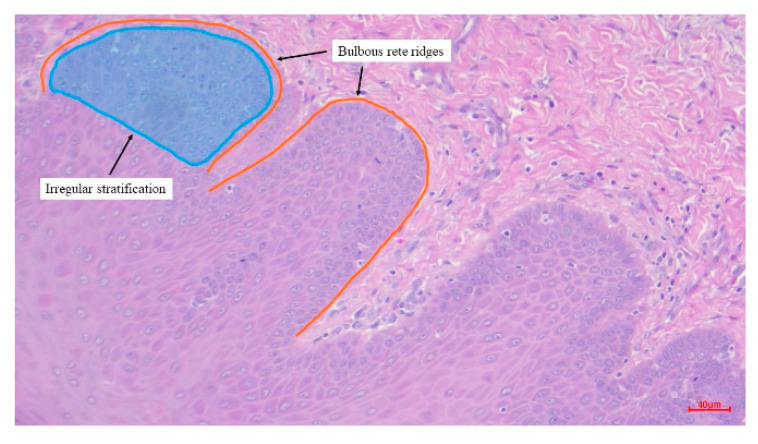
A histopathological image embodies irregular stratification and bulbous rete ridges as architectural dysplastic features.

There are seven types of OPMDs from the WHO classification. These include leukoplakia, erythroplakia, oral submucous fibrosis (OSMF), actinic cheilitis, palatal lesions in reverse smokers, lichen planus, and discoid lupus erythematosus [35]. Not all of these lesions will progress to squamous cell carcinoma. Only the lesions that contain dysplastic features can develop to oral cancer. Thus, dysplastic features must be detected and differentiated from these lesions. While the histopathology of squamous cell carcinoma is characterized by dysplasia and invasion of squamous epithelial cells into the basement membrane, the abnormalities go deeper than the epithelial layer and keratin pearls are sometimes found. In this study, we are interested in irregular stratification and bulbous rete ridges, which are considered architectural dysplastic features (Figure 2). Each has its own abnormal characteristics. In the epithelium, irregular stratification can be observed when cell arrangement is uneven. As can be seen, rete ridges are closely connected to the connective tissue. What we want to determine are bulbous rete ridges or the rete ridges that possess the shape of a broad base and a narrow top.

### 2.2. Dataset

**Figure 3:**
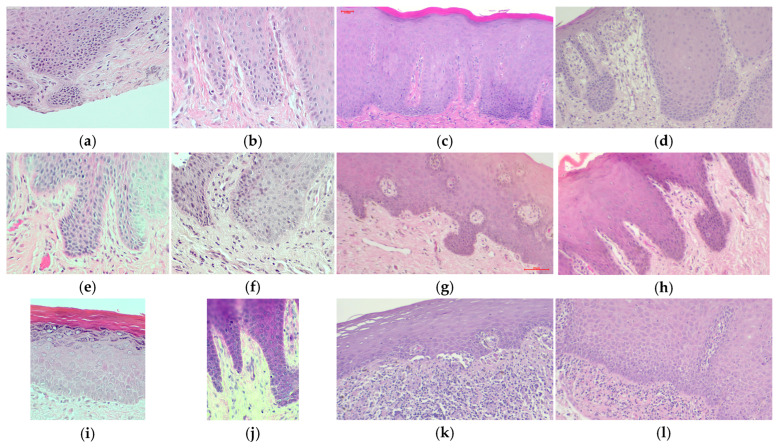
Sample microscopic (histologic) images in the dataset taken at 400× magnification. (**a**–**h**) are the images that contain irregular stratifications, bulbous rete ridges, or both. (**i**–**l**) are the normal images with no architectural dysplastic features.

To develop a dysplastic feature detector, we curated a dataset named Dysplasia-400×. Some sample images from the dataset are shown in Figure 3. It comprises multiple sources of 400× magnification images as described in Table 1.

The first and second sources were collected at the Department of Oral Biology and Diagnostic Sciences, Faculty of Dentistry, Chiang Mai University (Human Ethics Approval ID 66/2022), whereas the third source is courtesy of Tabassum Yesmin Rahman [35]. The dataset used in this research was diagnosed by three board-certified oral pathologists who followed the standard protocols of the Faculty of Dentistry, Chiang Mai University. In the process of annotation, the dataset was annotated by Dr. Nutchapon Chamusri, one of the diagnosing oral pathologists. Dr. Nutchapon Chamusri is an Assistant Professor in the Department of Oral Biology and Diagnostic Sciences at the Faculty of Dentistry, Chiang Mai University. He is a diplomate on the Thai Board of Oral Diagnostic Sciences (oral pathology) from the Dental Council of Thailand and the Royal College of Dental Surgeons of Thailand. He has expertise in oral pathology, oral cancers, oral potentially malignant disorders, and oral diagnostic sciences. His research focuses on the clinical and histopathological characteristics of oral lesions, pigmented oral mucosal disorders, and mitochondrial dynamics in odontogenic cysts and tumors. To ensure consistency and reliability, Dr. Chamusri conducted an intra-expert agreement process by revisiting and verifying his annotations, enhancing the dataset’s accuracy. These credentials and procedures ensure the annotations were performed with a high level of precision.

We manually selected 50 images with no OSCC from the Rahman’s OSCC dataset. These 50 images with no OSCC have different characteristics from our own collected images without OSCC. The reason for this is to increase the chances of correct detection of irregular stratification and bulbous rete ridges. The ground truth of regions that contain irregular stratification or bulbous rete ridges was manually delineated by a dental pathology expert. Although all image samples shared the same imaging procedure, it was unnecessary for them to be collected at the same time or camera setting. The image sizes can also be different even though they are from the same data source. Hematoxylin and eosin (H&E) staining, which is a common histochemical staining procedure, was utilized to dye different cell parts different colors before the images were taken with light microscopes.

One of the most challenging problems in dealing with this dataset is that the number of images that contain irregular stratification and bulbous rete ridges is relatively small. Furthermore, the number of bulbous rete ridges is less than half that of the irregular stratification.

From 208 images from the first two sources, 20 were segregated to form a blind set. Then, the other images would be used in training models. We opted to create a signature library (Table 2) from the 400× magnification images (excluding the blind set). All signatures had a size of 350 × 350 pixels. By framing the problem this way, we would have many more image samples for neural networks to learn from. Again, however, the problem of the small number of bulbous rete ridges remained.

In dealing with this imbalanced dataset, image augmentation was adopted to increase the number of bulbous rete ridge signatures only. Three image augmentation techniques were chosen to synthetically increase the number of signatures of bulbous rete ridges. We define the horizontal flip, the vertical flip, and the 180-degree rotation (which is equivalent to performing both the horizontal flip and the vertical flip together) thus:

(1) $$\left[\begin{array}{c} x^{\prime }\\ y^{\prime } \end{array}\right]=\left[\begin{array}{cc} -1 & 0\\ 0 & 1 \end{array}\right]\cdot \left[\begin{array}{c} x\\ y \end{array}\right]$$

(2) $$\left[\begin{array}{c} x^{\prime }\\ y^{\prime } \end{array}\right]=\left[\begin{array}{cc} 1 & 0\\ 0 & -1 \end{array}\right]\cdot \left[\begin{array}{c} x\\ y \end{array}\right]$$

(3) $$\left[\begin{array}{c} x^{\prime }\\ y^{\prime } \end{array}\right]=\left[\begin{array}{cc} -1 & 0\\ 0 & -1 \end{array}\right]\cdot \left[\begin{array}{c} x\\ y \end{array}\right]$$

respectively, where ${\left[\begin{array}{cc} x & y \end{array}\right]}^{T}$ is the source coordinate and ${\left[\begin{array}{cc} x^{\prime } & y^{\prime } \end{array}\right]}^{T}$ is the destination coordinate.

### 2.3. Image Processing

**Figure 4:**
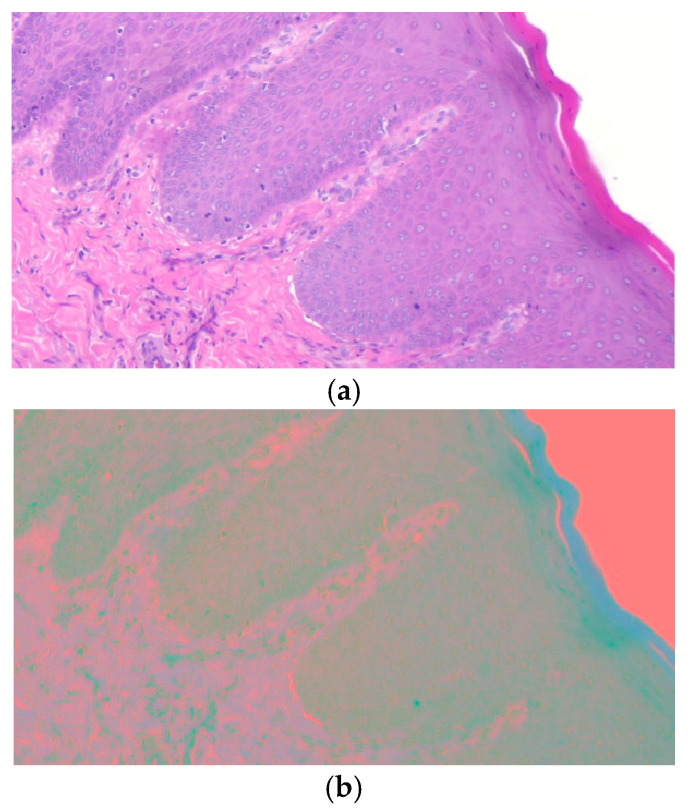
An original RGB microscopic image (**a**) and the corresponding YCbCr microscopic image (**b**) after applying (4).

Let us consider a discrete RGB image *I* of size *M* × *N.* Converting it from RGB to YCbCr can be carried out by changing each pixel I(m,n) with the following equation [36]:

(4) $$\left[\begin{array}{c} Y\\ C_{b}\\ C_{r} \end{array}\right]=\left[\begin{array}{c} 16\\ 128\\ 128 \end{array}\right]+\frac{1}{256}\left[\begin{array}{ccc} 65.738 & 129.057 & 25.064\\ -37.945 & -74.494 & 112.439\\ 112.439 & -94.154 & -18.285 \end{array}\right]\left[\begin{array}{c} R\\ G\\ B \end{array}\right]$$

where $I(m,n)={\left[\begin{array}{ccc} R & G & B \end{array}\right]}^{Τ}\in {\left[0,1\right]}^{3}$. Figure 4 illustrates a histopathological image sample before and after changing the color space. Every signature will be transformed from RGB color space into YCbCr color space.

## 3. Methodology

**Figure 5:**
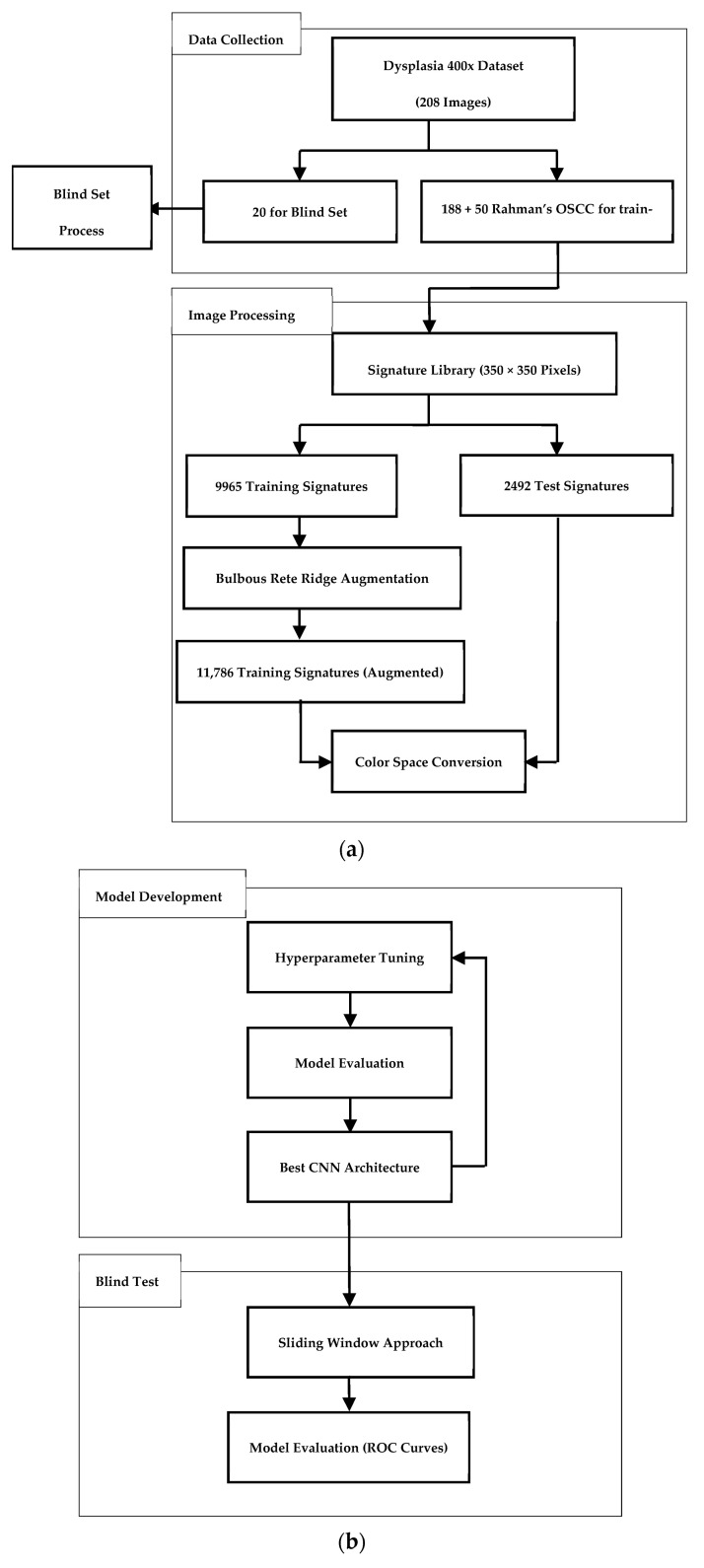
Overall methodology of the system: (**a**) preparing training and blind test data and (**b**) processes for training the model and testing the blind test data.

In this section, we describe the methodology employed to create a solution for detecting dysplasia in cell signatures (Figure 5). In practice, the trained model will be applied eventually via a sliding window approach to detect abnormalities in full multi-cell images.

### 3.1. Convolutional Neural Networks

Convolutional neural networks (CNNs) [37], a prevalent technique in contemporary computer vision, were selected to address the dysplastic features detection problem. A CNN architecture, just like typical neural networks, is represented by a sequence of three types of layers: (1) an input layer, (2) hidden layers, and (3) an output layer, but hidden layers are convolutional layers. We selected a CNN to act as a feature extractor. Its responsibility is to produce useful features given an input image by applying multiple convolutions successively. Finally, the extracted features, which usually have much lower dimensionality than its raw input, will be later used by a classifier.

A hidden layer of CNN, or convolution block, includes a convolution layer and a pooling layer. A convolution layer consists of a 2-dimensional convolution and an activation function. In this research, we adopt a generalized version of 2D convolution referred to as an à trous convolution (or so-called *l*-dilated convolution) [38]. Let us define $f:{\mathbb{Z}}^{2}\to \mathbb{R}$ be a discrete function which is either an image or a feature map. The kernel *w* is a discrete function of size *r* × *r* that maps to ℝ. À trous convolution operator is given thus:

(5) $$\left(f{⋆}_{l}w\right)(p)=\sum_{s+lt=p}f(s)w(t)$$

which has the resulting receptive field **p**. It enables us to vary the receptive fields by changing the dilation factor $l\in \left\{1,2,\ldots ,\min(M/2,N/2)\right\}$. When *l* = 1, it becomes a standard discrete 2D convolution. For instance, if *w* is a kernel of size 3 × 3 and *l* = 2, this setting gives us a receptive field of size 5 × 5, but it requires only 9 weights instead of 25 weights. Note that cross-correlations are actually used when it comes to the implementation; i.e., the kernels were not flipped.

Batch normalization (BN) [39] is a common technique in deep neural networks that normalizes the intermediate features. In this case, they are the outputs of convolution layers (after activation functions, before pooling layers). Batch normalization involves mini-batch normalizing, scaling, and shifting with the trainable parameters—the means and variances. Briefly, one of its effects is input distribution transformation and, hence, better model convergence.

One would have to use point-wise activation functions to introduce nonlinearity into our linear neural network to model nonlinear data with high complexity. The rectified linear unit, or ReLU [40], which is

(6) ϕ(x)=max(0,x)

is the nonlinear activation function applied to the output (feature maps) of the convolution layer prior to it.

To further reduce complexity in the later convolution layers, pooling layers are necessary. Pooling layers take feature maps and scale down their sizes. A maximum pooling operator [41] is chosen due to its simplicity and the fact that it maintains the highest value in the local area. With 2D maximum pooling with a kernel size of 2 × 2 and stride of 2, the feature maps are decreased to half of their original size.

In this study, the convolution block is composed of an *l*-dilated convolution, a point-wise convolution (which is the kernel of size 1 × 1), a batch normalization, an activation function, and a 2 × 2 maximum pooling.

The output of the last 2D convolutional layer is called *M* feature maps. Each feature map, $H^{\left(m\right)}=h_{i,j}^{\left(m\right)}$, has the same size, $F_{1}\times F_{2}$, for all m={1,2,…,M}. They will be fed as input into a classifier. Since an FCNN has been selected to be the classifier, we need a way to convert the feature maps in the form of 3D tensor into 1D vector. Instead of directly vectorizing (or flattening) the feature maps, global average pooling (GAP) appears to be a better choice and provides some regularization effect [42] because it can reduce the number of parameters by the magnitude of *F*_1_ × *F*_2_. The global average pooling layer’s output is a vector, $g\in {\mathbb{R}}^{M}$, given *M* feature maps. Each element of $g^{\left(m\right)}$ is obtained thus:

(7) $$g_{\mathrm{GAP}}^{\left(m\right)}=\frac{1}{\left|H^{\left(m\right)}\right|}\sum_{(i,j)\in H^{\left(m\right)}}h_{i,j}^{\left(m\right)}$$

where $\left|H^{\left(m\right)}\right|=F_{1}\times F_{2}$. It yields a vector of *M* dimensions, of which each element is the average of the corresponding feature map. GAP greatly reduces the complexity in bridging the output feature maps to a classifier.

In the experiment, we also explore a couple of alternatives to GAP as suggested in [43], i.e., global maximum pooling (GMP) and global log-sum-exp pooling (GLSEP). Among the global pooling operator variants, we need to empirically evaluate the performance of each variant and choose the best according to the accuracy metric. This is because one operator may perform better than another on a specific dataset. Global maximum pooling (GMP) can also be employed by, instead of taking the average over the features for each map, the maximum value from each map is obtained. The GMP is mathematically defined by:

(8) $$g_{\mathrm{GMP}}^{\left(m\right)}=\underset{(i,j)\in H^{\left(m\right)}}{\max}h_{i,j}^{\left(m\right)}$$

A smoother variant of GMP is global log-sum-exp pooling (GLSEP), which is obtained by substituting the maximum function in (8) with the log-sum-exp function. Therefore, the GLSEP can be alternatively written thus:

(9) $$g_{\mathrm{GLSEP}}^{\left(m\right)}=\log\sum_{(i,j)\in H^{\left(m\right)}}\exp\left(h_{i,j}^{\left(m\right)}\right)$$

GLSEP is not that strict compared to GMP, since the latter discards all values but the maximum value. On the other hand, GLSEP takes all values into account through the sum of exponentials which theoretically preserves more information. In other words, the higher the feature values, the more contributions to the output of GLSEP.

GMP is suitable for identifying the most significant or salient parts of the input due to its max operator. Meanwhile, GLSEP offers a balance between focusing on the most prominent features (like GMP) and averaging overall features (like GAP). In addition, it is fully differentiable, making it more suitable for gradient-based optimization methods [43].

### 3.2. Classifier

For the sake of simplicity, we also use a fully connected neural network (FCNN) and plug it in to the end of the CNN. This allows us to train a model holistically. Multiple layers of non-linear neurons compose the FCNN’s hidden layers.

A well-known regularization method named dropout [44] is also explored to help with the overfitting problem in our classifier. It randomly deactivates neurons in an FCNN hidden layer by creating masks *r_j_* ~ Bernoulli(*p*) for selecting nodes to be turned off. During training, if the *r_j_* are zero, then the corresponding nodes (which are not shut off) are multiplied by the scaler 1/(1 − *p*). During evaluation or inference, the dropout layers act as identity functions. In this work, we set *p* to be either 0.0 (no dropout) or 0.5 (about half of the neurons are deactivated).

Due to the fact that the task is to recognize two dysplastic features, we then designed FCNN architectures to have three output nodes which represent the class probabilities of *others*, *irregular stratification*, and *bulbous rete ridge* classes, respectively. The loss function for the whole neural network to optimize is accordingly defined to be the weighted cross-entropy loss function:

(10) $$l_{n}=-\sum_{c=1}^{C}w_{c}\log\left(\frac{\exp(x_{n,c})}{\sum_{i=1}^{C}\exp(x_{n,i})}\right)\cdot y_{n,c}$$

where *w_c_* is set to be inversely proportional to the number of samples of class *c*, *x_n_*_,*i*_ is the final network output of the *i*-th input sample, and *y_n_*_,*c*_ is the actual value. As mentioned earlier, the dataset has the problem of imbalanced class distributions. The weighted loss function will help mitigate the problem in the sense that the minority classes have higher impacts on adjusting weights and vice versa. Once the cost function is defined, backpropagation [45] plays a central role in adjusting the network’s weights over numerous iterations. The updated weights are those where they minimize the error defined in (10).

### 3.3. Training Techniques

When it comes to training CNNs, we also employed several training techniques to bring about training efficiency and stability as much as possible. The learning rates were set adaptively by starting from 10^−3^ and reducing them by half every 50 epochs. First, a mixture of 16-bit and 32-bit floating point precision was used to reduce the memory footprint and speed up the training time. In other words, the forward and backward passes are computed at half precision, and then the gradients are converted back to single precision. Second, even though we aim to obtain the probability mass function output from the softmax layer, the calculation of cross-entropy loss in (10) was carried out with the simplified mathematical form of the logarithm and exponential of unnormalized logits to attain numerical stability.

## 4. Experimental Results

*K*-fold cross-validation was selected as an evaluation method since the number of data samples is quite small. The typical train–test split is likely prone to population misrepresentation due to random sampling. The procedure is as follows. First, we split the data into *K* chunks. Second, the model is tested on the *k*th chunk after it is trained on the other three chunks. We then repeat this step for *k* = {1, …, *K*}. Here, *K* is set to four for all experiments; i.e., we will have four models trained on chunks of data for each architecture.

### 4.1. Signature Library

In designing neural network architectures, it is essential to check how the components influence the model performance. We thus tried to understand each component’s contribution empirically by changing the combinations of components and hyperparameters. After the networks are trained with four-fold cross-validation, they are evaluated on the test set for performance with detection metrics including accuracy, precision, recall, and F1 score, (Table 2).

Table 3 summarizes the ablation study by providing averages and the standard deviations of the accuracies of different architectures. *l_i_* denotes the dilated factor of the kernel size *w_i_* in the *i*-th convolutional layer in every convolution block. The number of hidden neurons of the *j*-th fully connected layer in the FCNN is represented by *h_j_*. The dropout rate *p* was set to be either 0 or 0.5. As can be seen from Architectures 1, 2, and 7, the most contributing factor here is the batch normalization added between the convolution layer and the activation function. Considering Architectures 2–4, GAP yields the best accuracy, while the accuracies of GMP and GLSEP are somewhat similar. Regarding the à trous convolutions, Architectures 2, 6, and 8 show that more dilated convolutions do not boost model performances. We did not make use of them by chaining the dilated convolutions in the exponential manner as suggested by the paper. Although they help with the larger receptive fields with a relatively smaller number of parameters, they also increase the complexity of the architectures. The accuracies of the different numbers of hidden neurons from Architectures 9, 11, and 12 indicate that 2048 seems to be the sweet spot. Interestingly, Architectures 12 and 16 have a subtle difference—between *h*_1_ = 2048 and *p* = 0, and *h*_1_ = 4096 and *p* = 0.5—that the dropout does not help much. Even the hidden neurons were dropped virtually by half. The color spaces also affect the performance as can be observed from Architectures 12 and 14.

In conclusion, the best architecture here is Architecture 12, which achieves 97.10% accuracy. This is the one trained on the second fold, as presented in Table 4.

### 4.2. Blind Test

After the result on the signature library is satisfied, we would like to observe how the model performs with the real-world use case, i.e., the original histopathological images. To do so, the sliding window approach is employed across a blind image. A stride of 10 pixels is used to reduce the computational cost, and, indeed, it comes with a trade-off between extensiveness and computational resources.

Recall that the output of the FCNN produces a probability mass function given an input image. Out of three output probabilities, the final decision needing to be made is which class the image patch belongs to out of three possible classes. The most common approach is thresholding on the probability values. Here, we chose the receiver operating characteristic (ROC) curve as a tool to measure the overall performance at different thresholding values for different classes. Moreover, the ROC curve can aid in determining optimal thresholding values of the class. Its x-axis represents false positives per image, i.e., the number of regions in which the model incorrectly predicts the positive class, but they actually are not. The y-axis indicates the probability of detection, which is how many positive regions are correctly detected.

**Figure 6:**
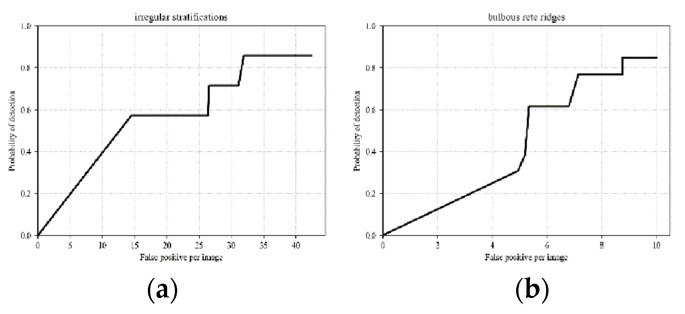
ROC curves of the 20 blind images for (**a**) irregular stratifications and (**b**) bulbous rete ridges.

**Figure 7:**
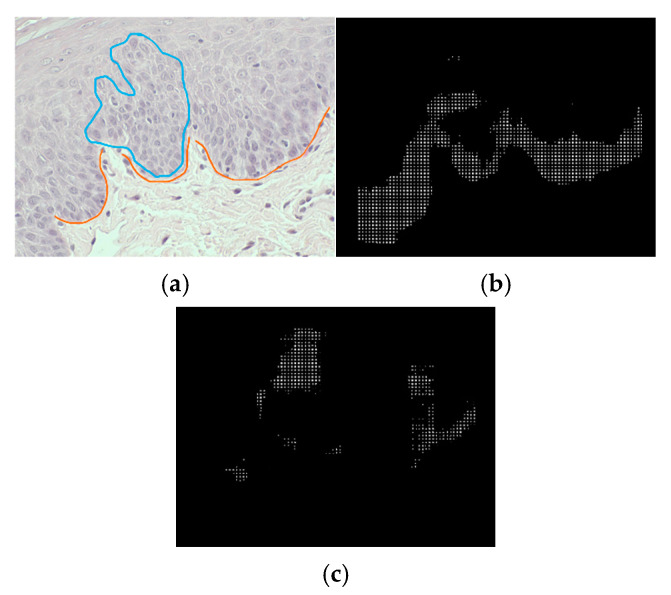
Examples of correct detection of (**a**) original image with labeled classes (blue line: irregular stratification, orange line: bulbous rete ridge), (**b**) bulbous rete ridge classes, and (**c**) irregular stratification classes.

**Figure 8:**
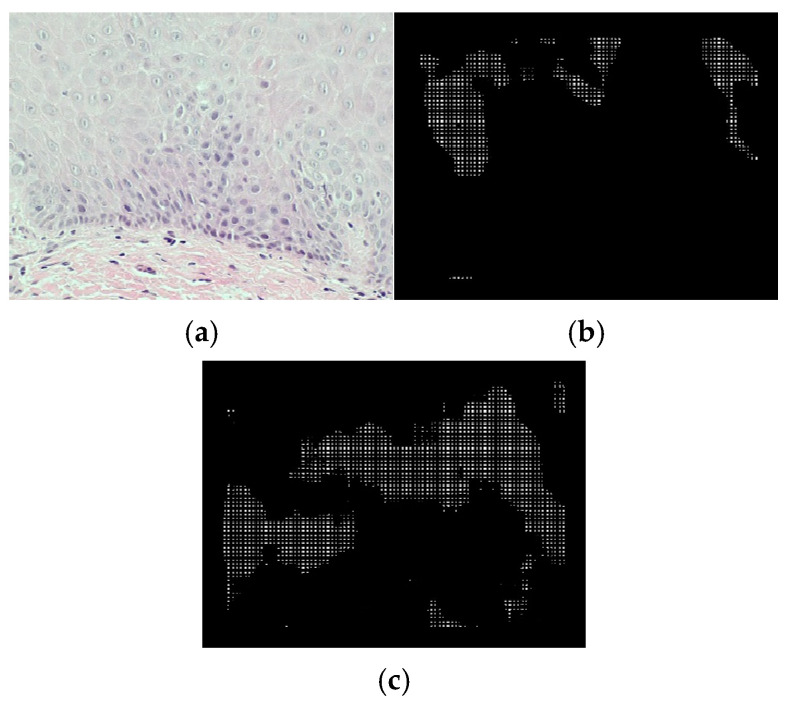
Examples of incorrect detection of (**a**) original image without either abnormality, (**b**) bulbous rete ridge classes, and (**c**) irregular stratification classes.

In Figure 6, the ROC curves of the irregular stratification and bulbous rete ridge classes are shown, respectively. For the irregular stratification class, the model attains a probability of detection up to 0.8571, and the lowest false positives per image, 31.9. The probability of detection for bulbous rete ridges is the highest, at 0.8462. The best-performing threshold of detecting bulbous rete ridges produces 8.65 false positives per image. Examples of correct and incorrect detection of both classes are shown in Figure 7 and Figure 8, respectively. In Figure 8, we can see that the area where the system detects bulbous rete ridge has similar shape to that of the real bulbous rete ridge class. The structure of the cell in the area that the system states that it is irregular stratification is also similar to the real irregular stratification.

## 5. Conclusions

In this paper, we propose a CNN-based model (Table 5) for detecting architectural dysplastic features from microscopic images. The proposed network trained on the signature library achieves the best accuracy, 97.52%, on the validation set. When it comes to the blind test with the sliding window approach, it can attain the best probabilities of detection of 0.8462 and 0.8571 for the irregular epithelial stratification and bulbous rete ridge classes, respectively. The goodness of the CNN-based model in this work is such that we do not need to perform segmentation on the whole slide image. We also do not need to perform feature extraction before carrying out classification.

However, when we implement this model on a large dataset, the accuracy might decrease because there might be some other conditions that affect the performance of the model, such as a change to the H&E stain color, the cell size from the microscope’s magnification, etc. Although we convert RGB into YCbCr, when H&E stain color changes, YCbCr also changes. This might affect the performance of the model. For future work, we need to reconsider some other features used in training the model. Also, if cell size changes, although CNN is a scale-invariant algorithm, when we apply the model to each sub-window in the blind test dataset, some parts of the cell might not be covered by that particular sub-window. Hence, the model will have incomplete information, and it might give an incorrect answer.

The proposed network is compact, as it only has around 1 million learnable parameters. Hence, it is easy to train and deploy with lower computational costs compared to the ones in the literature. Also, an ablation study is discussed for its impactful components and hyperparameters.

In the future, the proposed architecture can also be separated into two individual architectures, one for detecting irregular epithelial stratification and another for recognizing bulbous rete pegs. This design is likely to improve the performance of both classes, since we can also apply some post-processing to the final predictions. We will also explore cytological atypia and individual cell abnormalities. Although cytological atypia can have different characteristics from architectural dysplasia, we still can apply the same type of network with some modifications to tackle the problem.

## Figures and Tables

**Table 1:** Dysplasia-400× dataset.

Data Source	Irregular Stratification	Bulbous Rete Ridges	Blind Images	Training Images
Dysplasia	64	17	10	96
Normal	0	0	10	92
Rahman’s OSCC [15]	0	0	0	50

**Table 2:** Signature library.

Signature Library	Normal	Irregular Stratification	Bulbous Rete Ridges	Total Images
Training	6724	2634	607	9965
Training (augmentation)	6724	2634	2428	11,786
Test	1681	659	152	2492

**Table 3:** The impact of each component in the architecture.

#	Architecture	Accuracy (%)
1	*l*_1_ = 1, *l*_2_ = 1, *w*_1_ = 3, *w*_2_ = 1, No BN, GAP, *h*_1_ = 1024, *p* = 0	92.88 ± 0.59
2	*l*_1_ = 1, *l*_2_ = 1, *w*_1_ = 3, *w*_2_ = 1, BN, GAP, *h*_1_ = 1024, *p* = 0	96.88 ± 0.39
3	*l*_1_ = 1, *l*_2_ = 1, *w*_1_ = 3, *w*_2_ = 1, BN, GMP, *h*_1_ = 1024, *p* = 0	95.14 ± 0.78
4	*l*_1_ = 1, *l*_2_ = 1, *w*_1_ = 3, *w*_2_ = 1, BN, GLSEP, *h*_1_ = 1024, *p* = 0	95.08 ± 0.42
5	*l*_1_ = 2, *l*_2_ = 1, *w*_1_ = 3, *w*_2_ = 3, BN, GAP, *h*_1_ = 1024, *p* = 0	94.74 ± 0.94
6	*l*_1_ = 2, *l*_2_ = 1, *w*_1_ = 3, *w*_2_ = 1, BN, GAP, *h*_1_ = 1024, *p* = 0	96.57 ± 0.25
7	*l*_1_ = 2, *l*_2_ = 1, *w*_1_ = 3, *w*_2_ = 1, No BN, GAP, *h*_1_ = 1024, *p* = 0	92.10 ± 0.45
8	*l*_1_ = 3, *l*_2_ = 1, *w*_1_ = 3, *w*_2_ = 1, BN, GAP, *h*_1_ = 1024, *p* = 0	95.13 ± 0.32
9	*l*_1_ = 3, *l*_2_ = 2, *w*_1_ = 3, *w*_2_ = 1, BN, GAP, *h*_1_ = 1024, *p* = 0	95.27 ± 0.20
10	*l*_1_ = 1, *l*_2_ = 1, *w*_1_ = 3, *w*_2_ = 1, BN, GAP, *h*_1_ = 1024, *p* = 0.5	96.60 ± 0.33
11	*l*_1_ = 1, *l*_2_ = 1, *w*_1_ = 3, *w*_2_ = 1, BN, GAP, *h*_1_ = 512, *p* = 0	96.72 ± 0.48
12	*l*_1_ = 1, *l*_2_ = 1, *w*_1_ = 3, *w*_2_ = 1, BN, GAP, *h*_1_ = 2048, *p* = 0	97.10 ± 0.74
13	*l*_1_ = 1, *l*_2_ = 1, *w*_1_ = 3, *w*_2_ = 1, BN, GAP, *h*_1_ = 2048, *p* = 0.5	96.75 ± 0.72
14	*l*_1_ = 1, *l*_2_ = 1, *w*_1_ = 3, *w*_2_ = 1, BN, GAP, *h*_1_ = 2048, *p* = 0, RGB	96.79 ± 0.52
15	*l*_1_ = 1, *l*_2_ = 1, *w*_1_ = 3, *w*_2_ = 1, BN, GAP, *h*_1_ = 4096, *p* = 0	97.02 ± 0.61
16	*l*_1_ = 1, *l*_2_ = 1, *w*_1_ = 3, *w*_2_ = 1, BN, GAP, *h*_1_ = 4096, *p* = 0.5	96.88 ± 0.31

**Table 4:** Four-fold cross-validation results of the best network.

Fold	Accuracy (%)	F1 Score (%)	Precision (%)	Recall (%)
1	96.54%	95.76%	95.03%	96.54%
2	97.52%	96.48%	95.50%	97.52%
3	95.86%	95.30%	94.76%	95.86%
4	97.09%	96.64%	96.20%	97.09%

**Table 5:** The proposed network with 1,014,867 parameters.

Component	Output Shape	Model
Input	3 × 350 × 350	YCbCr image
Convolution layer (1)	16 × 348 × 348	2-dimensional convolutions [*l*_1_ = 1, 16 × (*w*_1_ = 3), BN, ReLU] [*l*_2_ = 1, 16 × (*w*_2_ = 1), BN, ReLU]
Pooling layer (1)	16 × 174 × 174	Maximum pooling (2 × 2)
Convolution layer (2)	32 × 172 × 172	2-dimensional convolutions [*l*_1_ = 1, 32 × (*w*_1_ = 3), BN, ReLU] [*l*_2_ = 1, 32 × (*w*_2_ = 1), BN, ReLU]
Pooling layer (2)	32 × 86 × 86	Maximum pooling (2 × 2)
Convolution layer (5)	256 × 18 × 18	2-dimensional convolutions [*l*_1_ = 1, 256 × (*w*_1_ = 3), BN, ReLU] [*l*_2_ = 1, 256 × (*w*_2_ = 1), BN, ReLU]
Pooling layer (5)	256 × 9 × 9	Maximum pooling (2 × 2)
CNN output	256 × 1	Global average pooling
Classifier	2048 × 1	Fully connected layer [*h*_1_ = 2048, ReLU, *p* = 0.0]
Output	3 × 1	[*h*_2_ = 3]

## Data Availability

Data available on request due to privacy restrictions.
